# Anti-BCMA novel therapies for multiple myeloma

**DOI:** 10.20517/cdr.2022.138

**Published:** 2023-03-22

**Authors:** Vincenzo Sammartano, Marta Franceschini, Sara Fredducci, Federico Caroni, Sara Ciofini, Paola Pacelli, Monica Bocchia, Alessandro Gozzetti

**Affiliations:** Department of Medicine, Surgery and Neuroscience, University of Siena, Azienda Ospedaliero Universitaria Senese, Siena 53100, Italy.; ^#^Authors share last co-authorship.

**Keywords:** Multiple myeloma, BCMA, belantamab, teclistamab, CART

## Abstract

Recent advances in multiple myeloma therapy have increased the depth of response and ultimately survivals; however, the prognosis remains poor. The BCMA antigen is highly expressed in myeloma cells, thus representing a target for novel therapies. Several agents that target BCMA through different mechanisms, including bispecific T cell engagers drug conjugated to antibody and CAR-T cells, are now available or under development. Immunotherapies targeting BCMA have shown good results in efficacy and safety in multiple myeloma patients previously treated with several lines of therapy. This review will discuss the recent development of anti-BCMA targeted treatments in myeloma, with a special focus on currently available agents.

## INTRODUCTION

Multiple myeloma (MM) is a clonal plasma cell (PC) disorder accounting for 10% of hematologic neoplasms^[1]^. Novel therapies such as proteasome inhibitors (PI), immunomodulatory drugs (IMiDs), and anti-CD38 monoclonal antibodies (mAbs), together with autologous stem cell transplant (ASCT), have significantly improved treatment outcomes of newly diagnosed MM patients with a continuous increase of the overall survival (OS) that today reaches a median of 10 years^[2-9]^. However, MM patients still do relapse and MM is considered an incurable disease^[10,11]^. In particular, triple-class refractory (refractory to PI, IMiDs, and anti-CD38 antibody) and penta-refractory (first and second-generation PIs, two generations of IMiDs, anti-CD38 antibody) patients have a median OS of 5.6 months, especially in the presence of high-risk cytogenetics (HR)^[12-20]^ or positive minimal residual disease^[21-26]^. Therefore, novel therapies, especially for relapsed/refractory myeloma patients (RRMM), are necessary^[25-26]^. BCMA is a B-cell maturation antigen highly expressed in myeloma cells, thus offering an encouraging potential target for novel treatments^[27-28]^.

### BCMA in multiple myeloma

BCMA, i.e., CD269 or TNFRSF17, is a TNF receptor superfamily 17 member, expressed on differentiated plasma cells and plasmablasts under physiological conditions and nearly on all MM tumor cells^[29-31]^. BCMA ligands include APRIL (A Proliferations-Inducing Ligand) and BAFF (B-cell activating factor) which are involved in the maturation and differentiation of PCs^[32]^. APRIL can bind to BCMA more avidly than to BAFF, and both can induce BCMA downstream signals to PI3K-PKB/Akt (i.e., phosphoinositide-3-kinase-protein kinase B/Akt), to RAS/MAPK (i.e., rat sarcoma/mitogen-activated protein kinase), and also to NF-κB (i.e., nuclear factor kappa-B), inducing increased plasma cells proliferation and survival^[31,33-37]^. Interestingly, PCs long-term survival in BCMA^-/-^ mice is defective, suggesting BCMA is crucial for a sustained humoral immune response^[38-39]^.

BCMA is overexpressed in myeloma PCs compared to normal ones, and its expression levels are elevated regardless of the stage of MGUS (monoclonal gammopathy of undetermined significance), SMM (smoldering multiple myeloma), and symptomatic MM^[40-41]^. Moreover, compared to healthy controls, APRIL and BAFF serum levels are 5-fold higher in myeloma patients. Recent studies showed that osteoclasts could be stimulated to produce more APRIL by MM cells, thus producing an immunosuppressive microenvironment^[31,35,42]^ Interestingly, MM cell proliferation can be reduced, in a mouse xenograft model, by a moAb directed against APRIL. Anti-BCMA immunotherapies, together with APRIL inhibition, can defeat MM-induced immunosuppressive microenvironment and intensify the ADCC (antibody-dependent cell-mediated cytotoxicity) against myeloma cells^[31,35,43]^.

sBCMA is the soluble form of BCMA, and it is produced by a γ-secretase acting on membrane BCMA^[44]^. sBCMA levels have been related to plasma cell infiltration in the bone marrow and may predict MM patients’ outcome. Indeed, some studies have shown that after MM treatment, the responsive patients resulted in lower sBCMA levels compared to patients with progressive disease^[45-47]^. Moreover, MGUS and SMM patients with higher levels of sBCMA showed a higher risk of progression to MM^[48-49]^. Thus, sBCMA might be used as a biomarker for disease progression and treatment response, allowing appropriate therapeutic management in case of drug resistance^[10,50]^. Additionally, one preliminary study in patients with non-secretory myeloma, for whom bone marrow aspirate and PET-CT scan are the only methods for disease monitoring, has shown that sBMCA levels correlate with the bone marrow PC infiltration, although this need to be confirmed^[45-46]^. Further studies are needed to validate sBCMA as a novel biomarker for MM and no approved diagnostic tool for measuring serum levels of sBCMA is available yet^[10]^.

Finally, sBCMA reduces BCMA expression on PCs’ surface, thus resulting in reduced efficacy of BCMA-targeted therapies and MM cells’ immune escape^[27]^. Additionally, authors showed that sBCMA at high levels might interfere with anti-BCMA therapy, thus reducing effective binding to MM cells^[51]^. Preclinical studies of γ-secretase inhibitor (GSI) have shown that it may decrease sBCMA levels and increase MM cells expressing surface BCMA, thereby improving response to BCMA chimeric antigen receptor T cell (CAR-T) therapy. Hence, the association of a GSI and BCMA-targeting therapy in MM patients is being evaluated in early-phase clinical trials^[2,52]^.

## BCMA-TARGETED TREATMENT IN MULTIPLE MYELOMA

**Figure 1:**
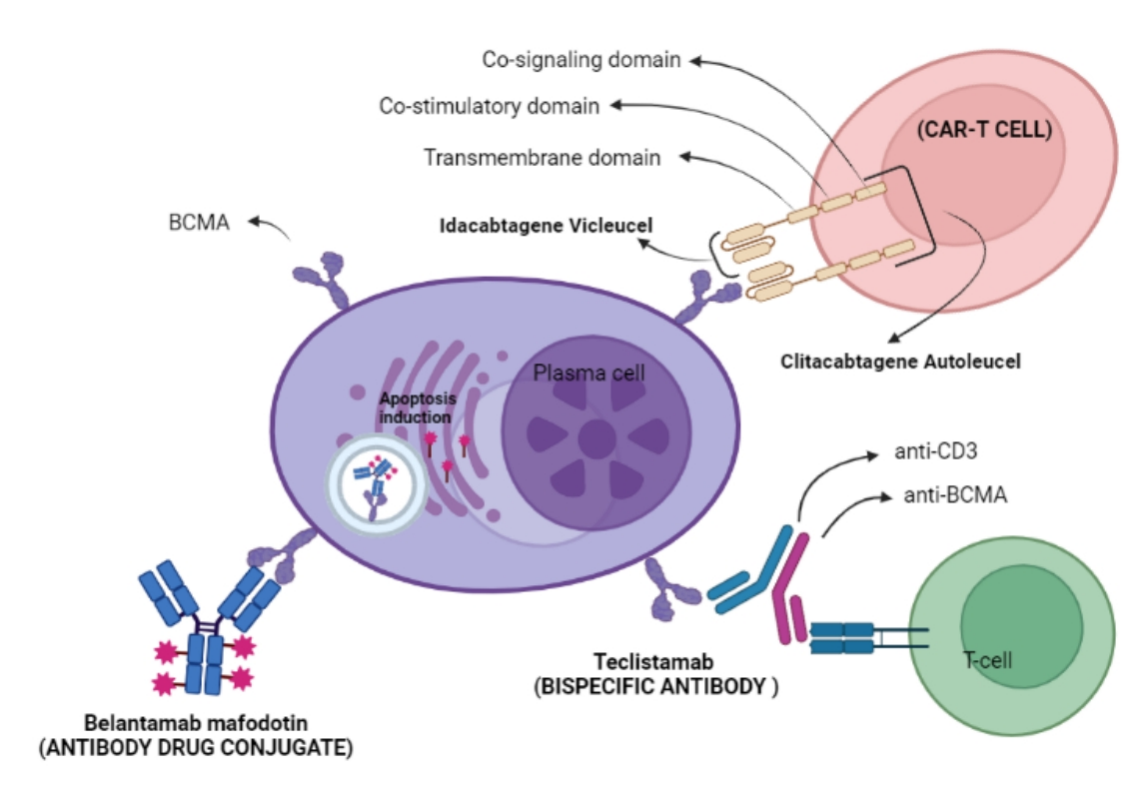
CAR: Chimeric antigen receptor.

The evidence that BCMA could be a suitable target for effective antitumor activity in preclinical studies led to the development of drugs targeting BCMA with several mechanisms [Figure 1]. Presently, BCMA-targeted therapies available are represented by: antibody-drug conjugates (ADCs), bispecific T cell engager (BiTEs), and chimeric antigen receptor (CAR)-T cells [Table 1]^[53,54]^.

**Table 1:** Characteristics of currently approved BCMA targeted agents

**Drug**	**Mechanism of action**	**Regimen of administration**	**Adverse effects**	ORR/CR (%)^*^	PFS (months)^*^
Belantamab (ADCs)	Monoclonal antibody conjugated with a cytotoxic agent	Intravenous (every 21 days)	Corneal toxicities Thrombocytopenia	31/3	2.8
Teclistamab (BITEs)	Fully humanized IgG4 bispecific antibody redirecting, CD3-positive T-cells to BCMA	Subcutaneous (weekly)	CRS ICANS Hematological toxicities	63/39	11.3
Idecabtagene Vicleucel (CAR-T)	BCMA targeted CAR-T incorporating anti-BCMA antibody costimulation domain, CD3ζ signaling domain	Single intravenous infusion	CRS ICANS Hematological toxicities	73/33	8.8
Ciltacabtagene Autoleucel (CAR-T)	BCMA-targeted CAR-T-cell product with two single anti-BCMA domain antibodies, CD3-ζ signaling domain costimulatory domain	Single intravenous infusion	CRS ICANS Hematological toxicities	97/67	Not reached

^*^Data from the registrational study; CR: complete response; ORR: overall response rate; PFS: progression-free survival.

### BCMA antibody drug conjugates

Antibody-drug conjugate (ADC) consists of a monoclonal antibody directed against a tumor- antigen and a cytotoxic agent inducing cell death (payload). ADC is internalized after binding to the related antigen on the tumor cell’s surface, then the linker is hydrolyzed inside of the lysosomes or endosomes and the payloads are released to cause cell death. ADCs can selectively target malignant cells with great efficiency on tumor cells and limited toxicities. Auristatin is a tubulin polymerase inhibitor used as a payload for MM^[55-60]^.

#### Belantamab Mafodotin (GSK2857916)

Belantamab mafodotin (Bel) is a humanized IgG1 ADC, first-in-class, originally approved by the FDA (US Food and Drug Administration) as monotherapy in relapsed myeloma patients treated with four prior therapies including a proteasome inhibitor, anti-CD38 monoclonal antibody, and an immunomodulatory agent^[61]^. Bel is formed by an antibody directed to BCMA and covalently linked to MMAF (the microtubule inhibitor monomethyl auristatin F)^[62]^. After binding to BCMA on MM plasma cell, Bel is internalized and MMAF is released, provoking cell-cycle arrest and apoptosis^[63]^. Other effects that seem to be mediated by Bel-binding BCMA are ADCC and antibody-dependent cellular phagocytosis (ADCP)^[64,65]^.

The multicenter phase I trial (DREAMM1) enrolled 73 RRMM patients. An ORR of 60% and PFS of 12 months were reported with acceptable toxicities. Corneal toxicity resulted in the most common non-hematologic side effect^[66,67]^. Subsequently, the phase II registrational study DREAMM2 enrolled 196 MM patients. The recommended dose was intravenous 2.5 mg/kg, Q3W. Reported ORR was 31%, with toxicities confirmed as manageable. A program was established to evaluate possible Keratopathy (Risk Evaluation and Mitigation Strategy, REMS) prior to drug administration^[68-71]^. Bel is currently being studied in different combination regimens in MM patients. The randomized, phase II study DREAMM4 is investigating Bel with pembrolizumab in patients with MM refractory to multiple lines of therapy. The DREAMM5 is testing Bel with other mAbs, such as isatuximab. The DREAMM-6 trial is exploring the combination of Bel, bortezomib, and dexamethasone *vs.* Bel, lenalidomide, and dexamethasone, while the DREAMM-7 and the DREAMM-8 studies are comparing Bel, bortezomib and dexamethasone *vs.* daratumumab, bortezomib and dexamethasone and Bel, pomalidomide and dexamethasone *vs.* pomalidomide, bortezomib and dexamethasone, respectively. Finally, the DREAMM-9 is testing Bel in the induction therapy in NDMM patients^[2,10,72,73]^. However, in November 2022, the FDA withdrew belantamab’s US marketing authorization as the DREAMM-3 trial (Bel *vs.* pomalidomide in combination with low-dose dexamethasone in RRMM) did not meet its primary endpoint of PFS (11.2 *vs.* 7 months, HR 1.03; 95%CI: 0.72-1.47). Sustainability could be a reason. Other Bel studies are ongoing, and results are awaited. Other studies including different anti-BCMA mAbs as well as ADCs targeting BCMA are ongoing^[74-76]^.

### BCMA bispecifics

BITEs are bispecific T cell engagers and represent a different modality of immunotherapy targeting BCMA. These agents are engineered proteins with two different antigen-binding fragments that bind to MM cells and T cells, thus creating an immunological synapse with direct plasma cell killing by T-cells^[77-79]^. The two common antigens involved are CD3 and CD16, and BCMA is the target of MM plasma cells. Many studies with BITEs utilizing BCMA showed great efficacy with moderate toxicity, such as CRS (cytokine release syndrome) and associated neurotoxicity syndrome (ICANS)^[80-82]^.

#### Teclistamab (JNJ-64007957)

In the MajesTEC-1 clinical trial, Teclistamab (Tec) was tested as an IgG4 bispecific antibody targeting CD3 on T-cells and BCMA in RRMM. Included patients were heavily pretreated, with two-thirds of them triple-class refractory and 30% penta-refractory. Tec was initially administered intravenously or subcutaneously in different cohorts, and safety was particularly improved, particularly in terms of reduced CRS, for the latter formulation. The recommended dose was 1500 μg/kg weekly subcutaneously, after two escalating doses of 60 and 300 μg/kg. The ORR was 63% (median PFS 11.3 months). CRS was observed in 72% of the patients (only 1 patient with a grade 3) and Il-6 inhibitor tocilizumab was needed in 37% of patients. The most common neurotoxicity reported was headache in 8% of the patients^[83-86]^. Those results were followed by Tec authorization for marketing as monotherapy in MM patients who showed disease progression during the last of three prior therapies, including a proteasome inhibitor, an immunomodulatory agent, and an anti-CD38 antibody^[87]^.

Others BITEs currently studied are Elranatamab (PF-06863135), ABBV-383, and alnuctamab (CC-93269). In addition, novel tri-specific agents that target BCMA are under preclinical evaluation and are demonstrating high clinical potential^[88-97]^.

### BCMA CAR-Ts

CART (Chimeric antigen receptor T) cell therapy act as cell-mediated immunotherapy. Briefly, after an in vitro gene transfer strategy, the patient’s T cells acquire the ability to can recognize tumor antigens (mostly used is BCMA) on MM plasma cells and thus destroy them. The CARs are formed by a receptor with an extracellular portion that binds to the antigen and an intracellular signaling domain. Moreover, the extracellular portion is formed by a single-chain variable fragment, i.e., scFv, connected to a transmembrane domain. CD28 is used as a costimulatory molecule. The final product results in a combination effect of mAbs and T cells cytotoxicit^[98-103]^. Leukapheresis of the patient’s T cells is the first step of CART generation. Thereafter, the scFv and costimulatory domains are introduced with a viral vector. Before reinfusion, patients usually receive a conditioning regimen of fludarabine and cyclophosphamide (a chemotherapy regimen used to achieve lymphodepletion) to decrease autologous T cells and permit CARTs proliferation^[104,105]^.

BCMA represents an ideal target for CAR-T therapy, and to date, two autologous BCMA-targeting CAR-Ts have been approved by the FDA, but several are being investigated in clinical trials^[106-108]^. BCMA is also being tested combined with CD19 for CAR-Ts multiple targeting^[109-111]^. A good efficacy has been demonstrated in early-phase clinical trials with bispecific CAR-Ts that target BCMA, CD19, or CD38^[112]^. Future alternative approaches could be represented by allogenic BCMA CAR-T cells or CAR-NK (CAR-natural killer), which are now investigated in early clinical trials^[113-121]^.

#### Ide-Cel, idecabtagene vicleucel ( bb2121)

Ide-Cel is a CAR-T of the second generation that targets BCMA. Ide-Cel includes a CD3ζ signaling domain and an scFv, a costimulating domain. A great efficacy has been shown in preclinical experiments against MM plasma cells. It is independent of levels of BCMA expression or sBCMA levels^[107]^. Ide-Cel showed an ORR of 85% and a median PFS of 11.8 months in heavily pretreated MM patients in a phase I study. Toxicities such as CRS and ICANS (mostly grade 1-2) were observed in 76% and 42% of patients, respectively^[122]^. The KarMMa phase II study was conducted in 128 MM patients who had previously received three or more lines of therapy, including a PI, an IMiD, and an anti-CD38 mAb. CAR-Ts infusion produced an ORR of 73%. Also, MRD negativity at 10^-5^ was seen in 26% of the patients (median PFS 8.8 months, 20.2 months when CR was achieved). Of note, when CAR-T was employed in high-risk disease patients (i.e., penta-refractory disease, extramedullary disease, or high-risk cytogenetic), results were confirmed. Toxicities were acceptable (CRS 84%, but only 7 patients (5%) with ≥ grade 3; ICANS 18%, with ≥ grade 3 in 4 (3%) MM patients^[123]^. Ide-Cel was approved by the US FDA thereafter for MM patients treated with four lines of therapy (comprehensive of a PI, an IMiD, and an anti-CD38 mAb)^[124]^. Ide-Cel is now being used in several trials to explore its efficacy in various scenario, including the use in first-line therapy or at early relapse^[125-127]^.

#### Cilta-cel ciltacabtagene autoleucel LCAR-B38M/JNJ-4528; Carvykti

Cilta-cel is a CAR-T-cell targeting BCMA with two antibodies to increase the binding avidity, a CD3-ζ signaling domain and a 4-1BB costimulatory domain^[109]^. In a recent phase I clinical trial, responses were high (ORR 88%) in RRMM patients after three or more prior lines of therapy (median PFS of 15 months). Toxicities were mostly grade 1-2 (CRS 90%, ICANS in 1 case)^[128]^. Subsequently, Cilta-cel was tested in 97 MM patients previously treated with multiple lines of therapy, with 40% of them being penta-refractory (CARTITUDE-1 trial). Interestingly, the response rate was quite high (> VGPR in 95%, MRD undetectable at 10^-5^ was achieved in 92%). Reported CRS and ICANS were similar to the previous study, but hematologic toxicities occurred more frequently (grade 3-4)^[129]^. Cilta-cel was then approved by FDA, in February 2022, for RRMM patients treated with > 4 prior lines of therapy including an anti-CD38 mAb, an IMiD, and a PI^[130]^. Recent ongoing phase III trials are CARTITUDE-2, evaluating cilta-cel efficacy and safety in different clinical settings in RRMM^[131]^; CARTITUDE-4, comparing Cilta-cel *vs.* pomalidomide, bortezomib and dexamethasone (PVd) *vs.* daratumumab, pomalidomide and dexamethasone (DPd) in RRMM; CARTITUDE-5, comparing bortezomib, lenalidomide, and dexamethasone (VRd) and Cilta-cel *vs.* VRd followed by lenalidomide and dexamethasone (Rd) therapy in transplant-ineligible patients MM at diagnosis^[132]^.

## BCMA, DRUG RESISTANCE, AND MM

While the efficacy and safety of BCMA-targeting agents have been demonstrated, data regarding drug resistance are also emerging, though the exact mechanisms of resistance towards these agents have not been fully understood^[133]^. Bone disease could be a reservoir for disease recurrence and a mechanism of resistance. Imaging is an important tool to detect residual disease outside the bone marrow or in extramedullary disease, although it is not known how BCMA antigen could be expressed on plasma cells outside the bone marrow. PET-CT is the gold standard technique to detect active disease and translated from lymphomas to MM^[134,135]^. In addition, whole body-MRI studies showed equal sensibility *vs.* PET-CT and can be used^[136]^. Downregulation of BCMA on PCs surface could be associated with resistance in a similar way as it has been described for CD19 and CD20 target therapies. Multi-targeted immunotherapies or the combination of BCMA targeting agents with γ-secretase inhibitors could overcome BCMA loss and both strategies are under investigation in clinical trials^[52]^.

Humoral and cellular immune responses could limit the persistence of BCMA CAR-T, leading to loss of efficacy and disease relapse. Alternative manufacturing processes, such as the application of human scFVs or the removal of the light-chain domain from the CAR antigen-binding domain, have been demonstrated to reduce CAR-T immunogenicity. In addition, BCMA CAR-T persistency could be increased by the addition of a phosphoinositide 3 kinase inhibitor during ex vivo culture to augment the memory-like T cells of the final product. Besides, allogenic CAR-T could overcome resistance related to T cell exhaustion which may be present in RRMM patients^[137-139]^.

Eventually, the tumor microenvironment is now considered to play a central role in promoting MM cell growth and has also been associated with drug resistance. Combination of BCMA targeting drugs with immunomodulatory agents could overcome this intrinsic mechanism of resistance, while trials are evaluating the next generation of armored CAR-T cells engineered to secret immunostimulatory cytokines or antibodies against inhibitory immune checkpoint receptors such as PD-1 and PD-L1.

## DISCUSSION

Despite novel therapeutic advantages in recent years, MM remains incurable. BCMA immunotherapies are a novel anti-MM therapeutic approach that holds promise to improve MM survival in the future. ADCs, BITEs, and CAR-T cells are the newest therapeutic options targeting BCMA. Early clinical trials showed great efficacy and safety even IN MM patients treated with > 4 prior therapy lines. Since comparative studies of anti-BCMA targeted therapies are still lacking, it is not yet known whether one of these classes of agents is superior to another; however, they all have unique toxicities and logistical challenges. ADC is an interesting and efficacious therapy, but corneal toxicities need further understanding. Bispecific antibodies are therapies that can be used with excellent clinical activity. Disadvantages of bispecific antibodies could be their short lifetime and the need to start treatment in a hospital setting, as severe CRS/ICANS side effects usually appear at the beginning of therapy.

CAR-T cells are also a great option, as clinical trials reported high response rates in heavily pretreated MM patients. The main drawbacks of CAR-T cells include manufacturing time and expenses, leukapheresis necessity, and use of chemotherapy and infusion in a hospital setting for toxicities management. In addition, a relevant mechanism of resistance could be represented by the limited CAR-T growth and contact with the adverse plasma cell myeloma microenvironment, thus resulting in limited therapeutic effects after one year^[140]^. To overcome these problems, new strategies are currently under investigation utilizing combos of drug agents with CAR-T, maintenance therapies after CAR-T, novel methods to extend CAR-T’s duration, and implementing CAR-T production^[141]^. Additionally, the combination of a checkpoint inhibitor with CAR-Ts is being tested as it may offer an advantage of reducing T cell downfall^[142]^.

The appropriate timing when to utilize a BCMA-targeted therapy is presently under investigation, with trials evaluating its role in earlier lines of therapy, including frontline. In fact, T cell-stimulating agents, such as CAR-T cells and BITEs, could probably produce deeper and longer responses if used at diagnosis or after only one or two lines of therapy, when MM patients are not heavily treated and may be at lower risk for T cell exhaustion.

In conclusion, therapies that target BCMA will play an important role in MM therapy, with the ambitious purpose of improving the cure rate; however, further investigations are still necessary to better define their real impact in clinical practice.

## DECLARATIONS

### Copyright

© The Author(s) 2023.
